# Deposition of Biocompatible Polymers by 3D Printing (FDM) on Titanium Alloy

**DOI:** 10.3390/polym14020235

**Published:** 2022-01-07

**Authors:** Dominika Grygier, Maciej Kujawa, Piotr Kowalewski

**Affiliations:** Faculty of Mechanical Engineering, Wroclaw University of Science and Technology, Wybrzeże Stanisława Wyspiańskiego 27, 50-370 Wrocław, Poland; dominika.grygier@pwr.edu.pl (D.G.); piotr.kowalewski@pwr.edu.pl (P.K.)

**Keywords:** FFF, PLA, PA, Ti-6Al-4V, friction, adhesion

## Abstract

Nowadays, the replacement of a hip joint is a standard surgical procedure. However, researchers have continuingly been trying to upgrade endoprostheses and make them more similar to natural joints. The use of 3D printing could be helpful in such cases, since 3D-printed elements could mimic the natural lubrication mechanism of the meniscus. In this paper, we propose a method to deposit plastics directly on titanium alloy using 3D printing (FDM). This procedure allows one to obtain endoprostheses that are more similar to natural joints, easier to manufacture and have fewer components. During the research, biocompatible polymers suitable for 3D FDM printing were used, namely polylactide (PLA) and polyamide (PA). The research included tensile and shear tests of metal–polymer bonds, friction coefficient measurements and microscopic observations. The friction coefficient measurements revealed that only PA was promising for endoprostheses (the friction coefficient for PLA was too high). The strength tests and microscopic observations showed that PLA and PA deposition by 3D FDM printing directly on Ti6Al4V titanium alloy is possible; however, the achieved bonding strength and repeatability of the process were unsatisfactory. Nevertheless, the benefits arising from application of this method mean that it is worthwhile to continue working on this issue.

## 1. Introduction

The replacement of a hip joint after osteoarthritis or fracture is nowadays a standard surgical procedure performed worldwide [1]. Despite this, there is an ongoing effort to improve the currently used hip endoprosthesis [2,3,4,5]. Researchers focus on creating endoprostheses that are structurally similar to the natural joint [6].

Sonntag et al. distinguished four approaches to the development of new endoprostheses [7]:

(a) The introduction of new materials;
(b) The application of a coating;
(c) The modifcation of the surface layer;
(d) The use of “soft” sliding materials.

The usage of “soft” sliding materials is the closest to a natural joint, where there is a soft tissue covering. Moreover, the use of “soft” sliding materials results in lower friction resistance and higher durability than with classic “hard” materials used in endoprostheses. The role of “soft” materials in the implant is fulfilled by plastics.

Recently, the popularity of 3D printing has significantly increased, meaning there is another technology for processing plastics. Additionally, 3D printing is also used in medicine. Most of the prints are less responsible elements such as models and surgical templates, although various types of implants are also printed [8,9,10]. Moreover, 3D printing provides the possibility to obtain personalized implants, which are mainly used during the replacement of bone defects, e.g., of the craniofacial region after injuries [11]. Additionally, 3D printing allows the reconstruction of the patient’s entire bone, e.g., the collarbone or the scapula, when the bone is attacked by cancer and requires complete removal [12]. Printed endoprostheses with individualized geometries are also used in challenging cases [13]. Ji and Guo described a case in which tumor removal required resection of a large portion of the pelvis and the entire hip joint [14]. The creation of a custom endoprosthesis made by 3D printing allowed for a more favourable solution for the patient. The authors of another paper indicated that by using 3D printing, the stiffness of the endoprosthesis can be adjusted to the stiffness of the bone tissue [15].

The 3D FDM prints are mainly tested for their mechanical properties [16,17,18,19]. However, papers analyzing the tribological properties of FDM 3D prints are appearing more frequently [20,21,22,23,24,25]. The influence of additives on the tribological properties of 3D prints has also been described [25,26,27,28]. Researchers have focused on the use of 3D prints in elements of machines. The tribological properties of FDM 3D prints intended for implant elements have rarely been discussed in the literature. This is due to the fact that such elements must be made of biocompatible plastics, and there are few biocompatible materials among those used in FDM 3D printing [29,30,31]. Moreover, the examinations must be performed in the presence of a medium that imitates synovial fluid, which is an additional complication [32,33,34].

Little information has been found on the 3D printing of sliding parts of endoprostheses using “soft” materials. Nevertheless, the published results encourage research to be conducted on this issue. Researchers have indicated that 3D prints are characterized by better tribological properties than parts produced by other methods. Borges et al. [35] compared the tribological properties of injected PCU (polycarbonate urethane) with that obtained by 3D FDM printing. Tribological investigations were conducted in the presence of fetal bovine serum. The printed samples exhibited 27% less wear than the injection-molded samples. The authors indicated that the printed specimens had higher porosity, meaning the serum was better absorbed. Therefore, the printed specimens acted similarly to the natural lubrication mechanism of the meniscus. The porosity of FDM prints has been studied by other authors, although in the context of mechanical properties rather than tribological properties [36,37,38]. Moreover, the porosity of 3D prints is seen as a great chance to produce scaffolds that promote tissue growth [39,40,41]. In terms of silicone (a material from a different group of plastics), no significant differences were found between the properties of a 3D printout and the sample manufactured by injection molding. In the mentioned studies, the tribological properties were not tested, although the absorption of water was examined and no significant differences were found [42].

“Soft” materials cannot be applied for the entire implant. They ought to work together with high-strength materials, since the implant needs to be fastened in the bone. As a result, implants consist of a metal core and a plastic “cap” that works frictionally with the metal. The plastic component must be bonded to the metal without the use of glue, since this kind of a substance could have a negative effect on the patient’s body. Consequently, the plastic components are attached to the metal ones using screws, bolts, or shaped connections (e.g., groove with wedge).

Metal–polymer combinations occur most frequently in automotive and aerospace applications. Most of the metal–polymer compositions used today are composites, for which the shape of the metal and polymer phases is random and the size of individual phases is in the nano- or micrometer range [43]. However, hybrid materials are also encountered. They show a clear distinction between the polymeric and metallic phases on the macroscale. Problem occur during the forming process of such hybrids, because both materials exhibit completely different mechanical properties [44,45]. The formation of such joints is based mainly on the mechanical “hooking” of metal elements in polymers; however, a method based on the formation of adhesive joints using increased temperature and pressure is also used [46,47,48,49,50].

There are proposals to use 3D FDM printing to create metal–polymer joints, although they are based on 3D printing by applying polymers to polymers, thereby creating a rivet that holds the metal part [51]. Other additive manufacturing methods were used to create the surface shape of the metallic material to enhance the bonding force [52]. No papers have been found describing the direct application of plastic to metal by 3D FDM printing.

When the plastic part is made using 3D printing, the plastic can be applied directly to the metal. This would simplify the manufacturing process and minimize the number of components used in the endoprosthesis. In addition, literature reports indicate that printed samples experience 27% less wear than injection molded samples [35]; thus, if 3D printing was used in endoprostheses, less wear could be expected (longer life of the component).

For these reasons, we decided to conduct research on 3D printing using FDM method on titanium alloy surfaces. Additionally, friction coefficients of plastic–titanium alloys were measured for cases of dry friction and in the presence of a lubricant.

## 2. Materials and Methods

The purpose of this study was to determine if it is possible to achieve a permanent plastic-to-metal bond by applying liquid plastic directly to metal using 3D FDM printing. Additionally, it was checked whether such applied 3D prints have a sufficiently low coefficient of friction to be used in endoprostheses. In order to carry out the research, it was necessary to decide which 3D printing method has the potential to apply plastic to metal. Moreover, the components consisting of plastic and metal are planned to be used in endoprostheses. Therefore, both the metallic material and plastic ought to be able to be applied in the human body. For this reason, the materials selected for the research had to be selected taking into account specific criteria, such as the biocompatibility. The deposition of plastics to metals by 3D printing is not yet practiced in research or industrial processes; therefore, a method for carrying out such an operation had to be developed. Moreover, the 3D printer needed to be adapted to this task. Appropriate adaptation was also required for the test stand, which was used to check the friction in the printed polymer–metal pair and the shear and tearing forces of the printed polymer–metal bond.

Summing up, preparation for the research included the following steps:

(a) Selection of 3D printing method;
(b) Selection of metallic material;
(c) Selection of biocompatible polymers;
(d) Development of a method for application of a thermoplastic polymer to metal;
(e) Adaptation of a 3D printer for application of a thermoplastic polymer to metal;
(f) Preparation of the test stand to measure the destructive forces of polymer–metal bonds.

This chapter describes in detail the preparation steps mentioned above.

### 2.1. 3D Printing Method

Currently, more than ten 3D printing methods can be distinguished [53]. The most important of these methods are:

- FDM (fused deposition modeling);
- SLA (stereolithography);
- SLS/SLM (selective laser sintering/selective laser melting);
- EBM (electron beam melting).

FDM is based on the deposition of molten material. Layer-by-layer the device applies a heated thermoplastic polymer, which cools down and hardens. In the SLA method, a photosensitive resin is used, which is hardened by laser light. The other methods use a material in the form of a powder, with differences in the way the grains are fused. In SLS, the grains are sintered together by laser, while in SLM the powder is completely melted and solidifies after cooling. In the case of EBM, the melting is achieved using an electron beam.

Among the mentioned 3D printing methods, the FDM method is currently the most widespread, since it requires the least complicated and consequently the cheapest equipment [54]. In order to make the results useful to more 3D printer users, it was decided to use the FDM method to attempt to make prints on metal.

### 2.2. Metallic Material

Among all metallic biomaterials, titanium and its alloys are currently the best materials for various types of implants. Titanium has fine mechanical properties and very good corrosion resistance and biocompatibility. Additionally, it has the lowest specific gravity and Young’s modulus as compared to austenitic steels and cobalt alloys. The very good biotolerance of titanium in the environments of living organisms causes the occurrence of the process of osteointegration (fusion of bone tissue with the titanium surface of the implant). This element has a high affinity for oxygen, thanks to which a passive layer of TiO_2_ easily forms on its surface, protecting it against corrosion. Due to the complex manufacturing processes, making an implant from a titanium alloy is associated with high costs.

The classification of titanium alloys is based on the structural criterion in the equilibrium state. Pure titanium exists in two allotropic varieties: α (at transformation temperatures lower than 882 °C) and β (at transformation temperatures higher than 882 °C). By adding alloying additives and using appropriately selected thermomechanical processes, titanium alloys used in bioengineering can be divided into three groups: single-phase α alloys, dual-phase α + β alloys and single-phase β alloys.

The metal on which the plastic was printed was titanium alloy Ti6Al4V. It is one of the most popular metals used in orthopaedic synovial joint replacements (knee or hip joints), as it is characterized by low density, high strength, superior corrosion resistance and excellent biocompatibility [55]. It is a two-phase titanium alloy, with aluminium strengthening the α phase and reducing the density of the alloy and vanadium stabilizing the β phase. The tensile strength of the alloy is about 860 MPa and the yield strength is about 780 MPa. The elongation ought to be within 10%. Heat treatments such as quenching and tempering or supersaturation and ageing can change the phase morphology and increase the mechanical properties.

Titanium alloys are used to produce joint and knee endoprostheses, intramedullary nails, plates, bone screws and various prosthetic products (bridges), and are used in cardiac surgery and surgical cardiology. Ti6Al4V alloy is suitable for processing by 3D EBM printing, and this method is used to make implants for skull reconstruction, among other uses [56,57,58].

### 2.3. Plastic Materials

A wide range of thermoplastic polymers are used in FDM 3D printing. The group of plastics used in this method contains more than a dozen materials. Moreover, more thermoplastics, as well as composites based on their matrices, are continually proposed for use in 3D FDM printing [59,60]. However, we conducted research on the methods used for applying plastic by FDM 3D printing with the intention of using manufactured components in implants. Therefore, the plastic used ought to be biocompatible, which means that it will not have a toxic effect on the human body, will not affect the immune system and will not have a destructive effect on the blood. Only a few of the many materials used in FDM 3D printing are biocompatible, namely polylactide (PLA), polyamide (PA) and polyetheretherketone (PEEK).

PLA is one of the most widely used materials in FDM technology and is used as a biocompatible and biodegradable material introduced into the body [61].

PA is more challenging than the most popular materials used in FDM 3D printing; however, obtaining good quality prints is not as hard as with PEEK. PA filaments are available and manufacturers provide recommended printing process parameters.

PEEK is often used for implants. Such 3D prints made from PEEK replace ribs [62] or serve as vertebral stabilizers in the spine [63]. Additionally, 3D FDM printing with PEEK is possible, although it is a difficult process and requires a printer with above-standard parameters [63].

Ultra-high molecular weight polyethylene PE-UHMW is worth mentioning too, since it is a polymer that is widely used in endoprostheses. Due to the fact that it is a thermoplastic, it would seem that it can be used in FDM. Unfortunately, this material is characterized by an almost zero MFI melt flow index, which eliminates the possibility of extruding this material and its use with the FDM method. Only high-density polyethylene PE-HD [62], which is a material from the same group as PE-UHMW (however, with much weaker mechanical properties), can be successfully 3D-printed using FDM. Currently, PE-UHMW is successfully used only with the SLS printing method [64].

No papers regarding the deposition of plastic layers on titanium alloy by FDM printing were found. As a result, no experience from other studies was gathered and work was carried out on the deposition of plastics by 3D printing from the very beginning. Hence, it was decided that the work would begin with tests with PLA and PA, which are materials that are available and unproblematic for FDM 3D printing. PEEK, which is a difficult material for FDM 3D printing, was excluded from this initial stage of research. Positive results from the verification tests would allow the technology for manufacturing metal–polymer components using advanced polymeric materials such as PEEK to be developed in the next stage.

Finally, two materials were used to make the prints: polylactide (PLA) and polyamide (PA). Prints were made on a Zortrax M300 Dual printer. The filaments used were also from the same manufacturer, and their trade names were Z-PLA (polylactide) and Z-Nylon (polyamide). It was decided to use materials from Zortrax, since in FDM the printing process parameters are very important. The printer software has in its database parameters dedicated to these materials, which significantly increases the chances of a successful printing process. Moreover, Zortrax provides test results for their materials, providing the values for their most important properties (Table 1).

### 2.4. Surface Roughness

A very important factor in the formation of plastic–metal bonds is the roughness of the metal surface. An analysis of the influence of surface roughness on the interactions between the bonded surfaces showed two mechanisms of adhesion force formation. These interactions were related to:

- Adhesive interactions;
- Mechanical interactions.

**Figure 1:**
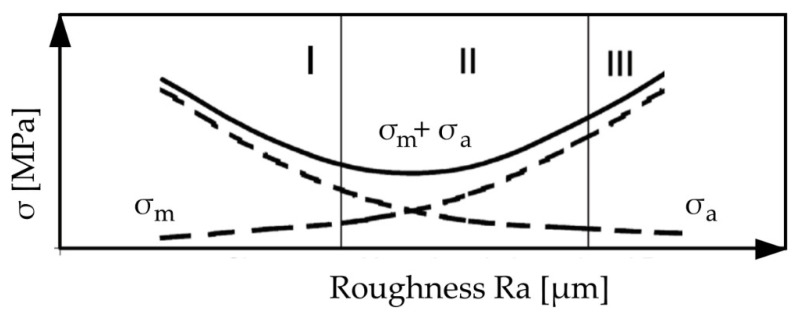
Influence of surface roughness on the failure stress at rupture of a metal–polymer bond.

In terms of very smooth surfaces (zone I in Figure 1), adhesion of a polymer surface to the surface of metal element plays the dominating role in the interactions. This results in relatively high stress appearing during polymer–metal separation (σ_a_). The phenomenon of adhesion is an effect of an increase in the actual contact surface, bringing the particles of joined surfaces closer to the range of second-order interactions.

As the roughness increases, the value of the joining force might decrease (the adhesion decreases—zone II in Figure 1). In terms of large roughness, the mechanical hooking of surfaces increases in its contribution, which increases the stress appearing at the polymer–metal separation zone (σ_m_) (zone IIII in Figure 1).

The influence of surface roughness on the stress value during the separation of polymer from metal (σ_a_ + σ_m_) might occur as shown in Figure 1.

The effect of surface roughness on the bonding strength of metal–polymer components ought to be investigated. Two different surface states are recommended to test metal–polymer components. The surface roughness values of the Ti6Al4V alloy with the following values of the Ra parameter ought to be used:

- Ra < 0.5 µm (smooth surface);
- Ra > 5 µm (rough surface).

The use of such roughness values will allow one to verify the influence of this parameter on the binding force during the tests. The values of roughness parameters are presented in Table 2. R_a_ is arithmetical mean deviation of the assessed profile. R_z_ is the maximum peak-to-valley height ratio of the profile (within a single sampling length). The value of Rz is averaged over the assessment length. R_a_ and R_z_ are the most popular roughness parameters used globally.

### 2.5. Shape of the Bond

**Figure 2:**
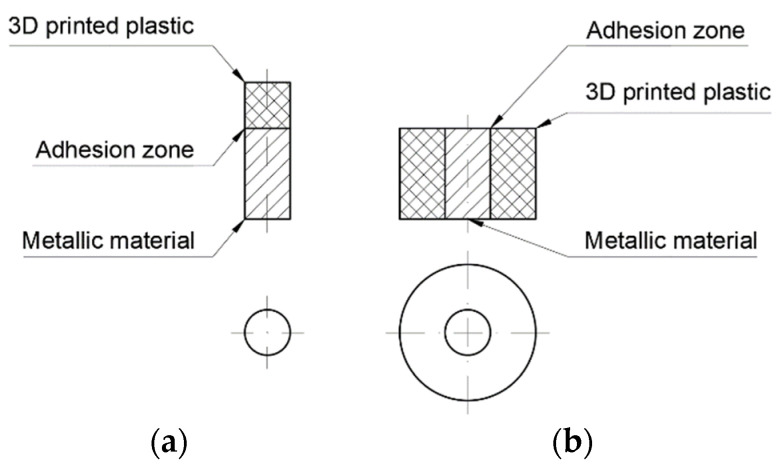
Geometric variants of the printing of metal–polymer components: (**a**) printing with a flat surface (simple method); (**b**) printing with a complex surface (method requiring software modifications).

The creation of metal–polymer components by 3D FDM printing can be accomplished in two ways:

(1) The metal component can be placed in the printer workspace under the printer head (Figure 2a). This will allow the printer to apply plastic with standard movements without the danger of collision with the sample;
(2) The printer’s nozzle can move next to the sample, meaning a collision with the sample may occur. In this case, it is necessary to develop custom software to determine the movements, so as to avoid collision with the specimen (Figure 2b).

The first case gives the possibility of obtaining components without any changes in software. However, this simplifies the surface-shaping possibilities between the metal and polymer components.

In the second case, more shapes are possible; thus, not only the adhesion force but also shape connection can be used to obtain the highest possible binding force between two materials. However, this variant requires the development of software by taking into account the presence of the metal component in the working field of the 3D printer. Additionally, 3D printers use various types of software. Some developers make source code available and some do not; thus, not all software can be changed. Therefore, the choice of this option significantly limits the number of end users who can use this method.

The variant shown in Figure 2a was used to produce metal–polymer components. It is less demanding to implement and is based on the use of already existing 3D printers. As a result, it is available to a significantly wider group of 3D printer users.

### 2.6. Method of Manufacturing Metal–Polymer Components

The creation of metal–polymer components by 3D FDM printing can be accomplished in two ways. In the FDM method, the first layer of molten plastic is applied to the printer’s bed. In order to achieve successful printing, the material must adhere to the working table throughout the process. After the printing is completed, the finished element must be separated from the table without damaging the machine or the printout. Glass is most often used on the bed, since when it is heated up it keeps the printout on the table and when it is cooled down the printout can be separated from it. We did not manage to find papers describing the application of the material using 3D FDM printers and involving its permanent maintenance on the metal surface, meaning we had to create our own method.

**Figure 3:**
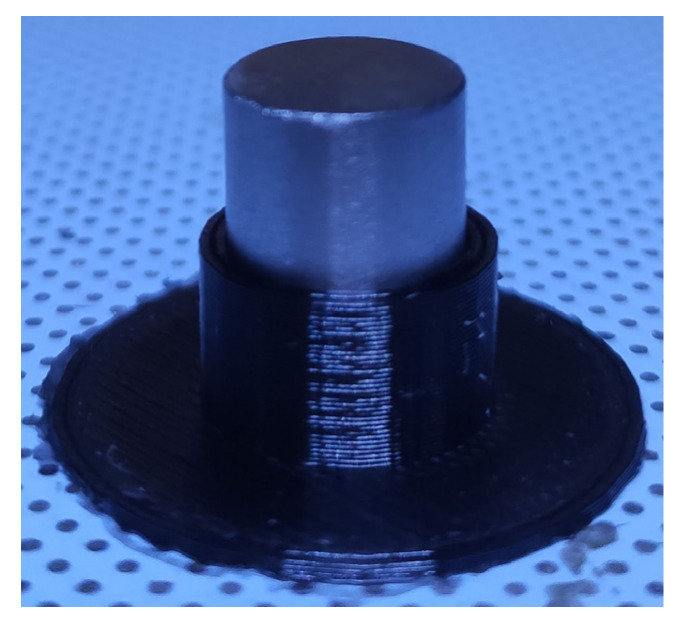
The 3D-printed sample holder that maintained the sample on the heated printer bed.

Prints were made using a Zortrax M300 Dual printer. The first attempts at printing consisted of printing a holder for a titanium alloy sample on the printer bed (Figure 3). Most 3D printer control programs allow the user to start depositing plastic at a selected height. In such cases, after entering the height of the sample, the printer will deposit the plastic to the metal without collision.

The sample was heated using a heated printer bed. The maximum temperature was 100 °C. Unfortunately, attempts to deposit PA and PLA failed—the plastics did not adhere to the titanium alloy. The application of plastic coatings is practiced in the industry (usually where chemical resistance is required). Coatings of this kind usually replace rubber ones because they do not age as quickly. Both the plastic being applied and the metal part must have temperatures above the softening temperature of the plastic. The printer bed could only heat up to 100 °C, meaning an additional heater was required.

**Figure 4:**
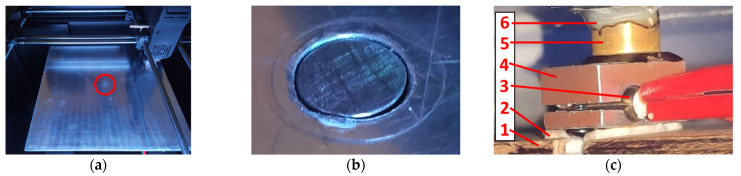
Replacement of printer bed for heating of a titanium alloy sample to 260 °C: (**a**) aluminum alloy plate with a centrally located sample hole (circled in red); (**b**) sample placed in the socket; (**c**) heating system located under the plate: 1—beam fixing the heating system; 2—thermal insulation; 3—heater with temperature sensor; 4—heating block; 5—sleeve fixing the sample; 6—thermal insulation made of epoxy resin.

An aluminum alloy plate was mounted instead of the printer bed (Figure 4a). A hole was made in the center of the plate where the sample was placed (Figure 4b). The printer was equipped with a capacitive sensor and automatically adjusted the slope of the plate and its distance from the nozzle. Under the plate was a bar on which a heater with a sleeve holding the sample was located (Figure 4c). The aluminum alloy heating block contained the heater and temperature sensor. A sleeve holding the sample and transferring heat to it was placed on the block. A layer of epoxy resin was applied between the aluminum alloy plate and the sleeve to provide insulation and prevent the plate from receiving too much heat from the heater. The heater was controlled using a transistor. This allowed the heating block to be maintained at any temperature up to 260 °C with an accuracy of ±1 °C. The installation of the additional heater is a low-complexity, low-cost operation that requires components that are easily available.

**Figure 5:**
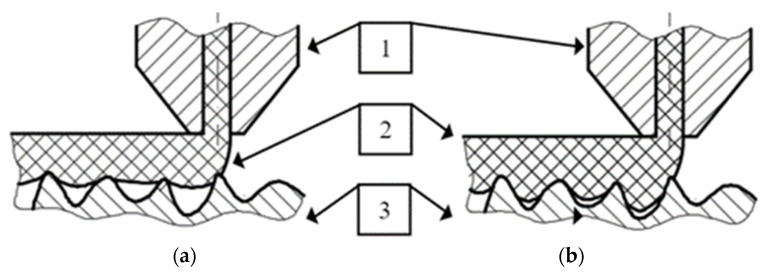
Application of the first layer: (**a**) standard flow; (**b**) increased flow; 1—printer nozzle; 2—first layer; 3—substrate.

Maintaining the print on the printer bed is crucial during the FDM process. The authors of this paper conducted research to determine which parameters are important to keep the print on the printer bed [67]. The research showed that increasing the flow of plastic is crucial. This allows the material to be “pushed” into the surface and to better fill its irregularities (Figure 5).

**Figure 6:**
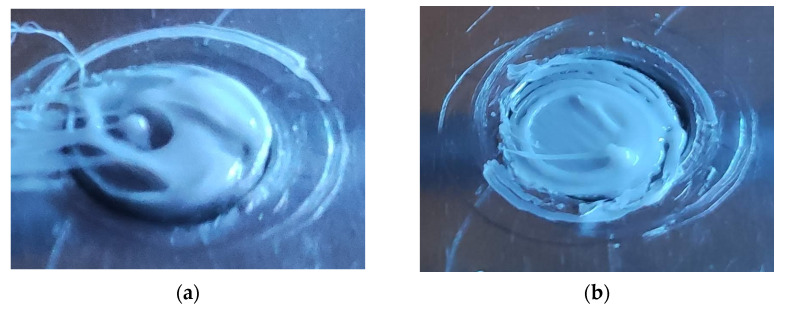
First layer applied on the titanium alloy sample: (**a**) increased flow; (**b**) standard flow.

During attempts to deposit plastic on the titanium alloy, the authors took advantage of the experience from the research mentioned above. Unfortunately, when the flow was increased, the nozzle dragged the material. As a result, the initial layer on the sample surface was uneven and did not cover the entire metal surface (Figure 6a). Significantly better coverage was achieved with the standard flow (Figure 6b). When printing, the parameters recommended by Zortrax obtained from the software database were used. The only change was that the application speed of the first layer was reduced by 50% to reduce the drag of the plastic.

The samples were heated to a temperature equal to the printer nozzle temperature. The nozzle temperature was given by the material manufacturer. At this temperature, the material has good plasticity. The printing procedure included:

1. De-greasing the sample;
2. Warming up the sample;
3. Applying three layers of plastic;
4. Stopping printing, turning off sample heating and cooling the sample to 60 °C (PLA) or 160 °C (PA);
5. Resuming printing.

**Figure 7:**
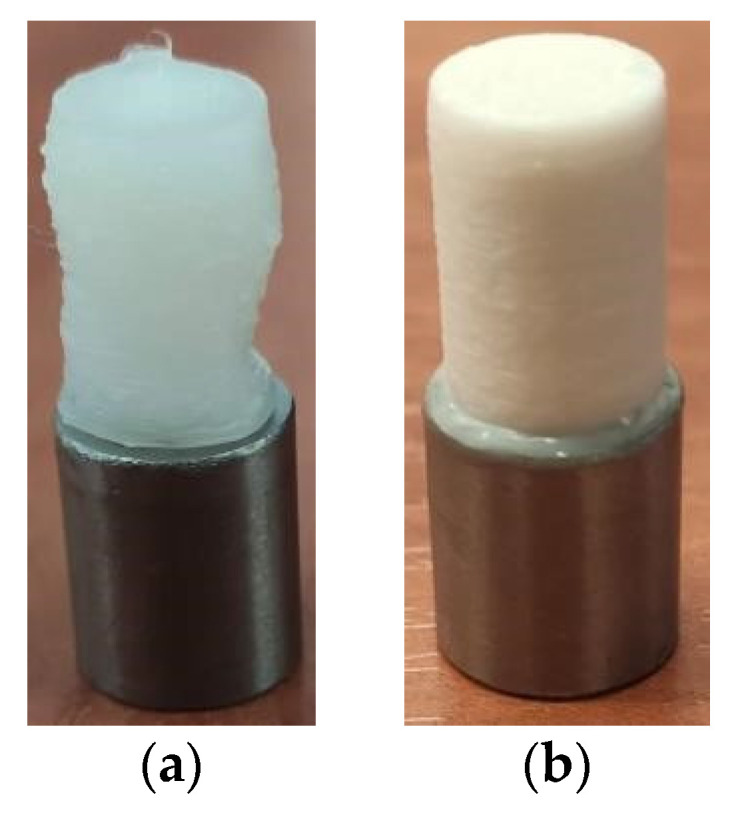
Obtained prints: (**a**) deformed PA overprint on a sample that was not cooled down after three layers were applied; (**b**) correctly printed PLA overprint.

When the printing was not stopped and the sample was not cooled, the already applied material was too plastic in nature to hold the desired shape (Figure 7a). The nozzle applying the material dragged the plastic, and as a result the print was significantly deformed. Cooling the sample after applying three layers caused better print accuracy (Figure 7b). The printing process took about 12 min (without waiting for the sample to heat up and then to cool down).

Researchers agree that the parameters for the 3D FDM printing process have a significant influence on the properties of the printed element [68,69,70,71,72,73]. Due to the fact that the print parameters are crucial, the parameters used for depositing plastics with 3D FDM printing to metal are presented in Table 3.

### 2.7. Testing of Metal–Polymer Bond

Metal–polymer specimens were subjected to strength and tribological tests. The aim of strength testing was to determine shear and normal stresses causing rupture of the plastic–titanium alloy bond. Tribological investigations provided data on the coefficient of friction for pairs consisting of printed specimens. The results of strength and tribological tests allowed us to determine whether metal–polymer components could be useful in endoprostheses.

Materials applied to various types of substrates (e.g., coatings) are most often tested in accordance with the ISO 4624 standard. The method described in this standard consists of sticking test dollies to the coating and removing them. The use of this method requires a coating with a relatively large surface area. In addition, the coating ought to be removed all around the test dolly before pulling it off.

Increasing the surface area of the deposited material might cause difficulties in the manufacturing of specimens. The metal element required heating during the deposition of plastic. Increasing its surface area would require the use of a heating system with a greater power, which would make it more difficult to maintain a precisely set temperature. Moreover, the coatings turned out to be poorly bonded to the substrate. The need to remove the material around the test dollies would probably detach the coating and make it impossible to test it.

Moreover, the ISO 4624 standard describes the removal of coatings, and when carrying out the shear strength tests, alternative methods (ISO 2407 or ISO 16276-2) ought to be used. Due to the above-mentioned factors, we decided to use our own test stand to detach plastic samples deposited on metal. The test stand described below was inspired by the guidelines from ISO 4624.

#### 2.7.1. Tensile Strength Tests

**Figure 8:**
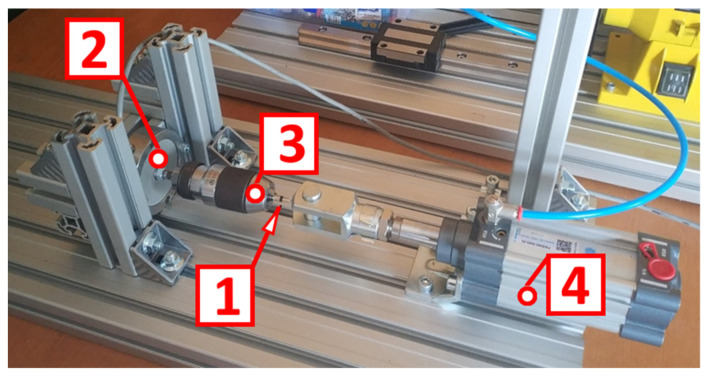
Test stand used to determine the tensile strength of metal–polymer joints: 1—specimen; 2—force sensor; 3—drill chuck; 4—pneumatic actuator.

The test stand shown in Figure 8 was used to test the tensile strength of each joint.

One end of the test component sample (1) was rigidly fixed in a drill chuck (3), which was screwed to a force sensor (2). The other end was pulled by a flexible wire attached to a pneumatic cylinder (4). The gradual increase in pneumatic pressure increased the normal stress value in the metal–polymer joint until rupture. The highest stress that was transmitted through the component was taken as the strength of the bond.

#### 2.7.2. Shear Strength Tests

**Figure 9:**
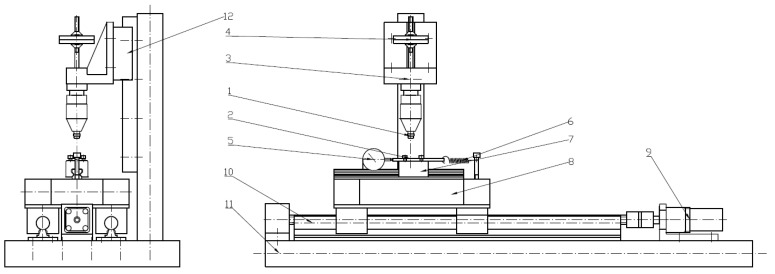
Test stand: 1—specimen; 2—counter-specimen (metal plate); 3—specimen clamping assembly; 4—weights; 5—force sensor; 6—stop spring; 7—upper carriage; 8—lower carriage; 9—stepper motor with gearbox; 10—linear guide for the lower carriage; 11—base; 12—linear guide for specimen clamping.

**Figure 10:**
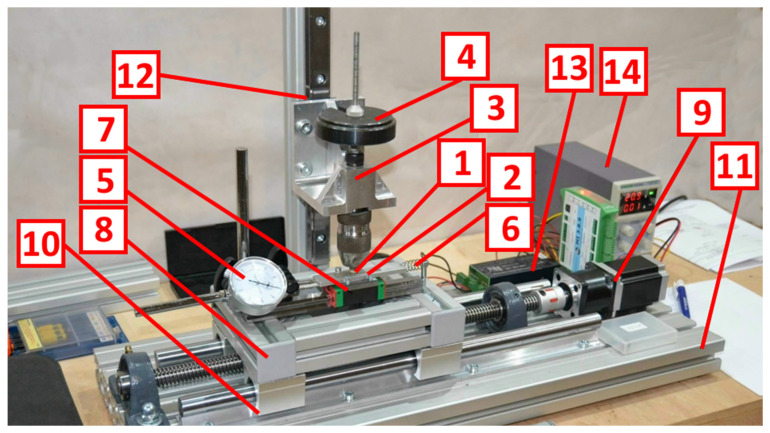
Test stand: 1—specimen; 2—counter-specimen (metal plate); 3—specimen clamping assembly; 4—weights; 5—force sensor; 6—stop spring; 7—upper carriage; 8—lower carriage; 9—stepper motor with gearbox; 10—linear guide for the lower carriage; 11—base; 12—linear guide for specimen clamping; 13—stepper motor controller; 14—power supply unit.

A single test stand was used for shear strength determination and tribological testing. For shear strength measurements, a force sensor was mounted on the device to record the shear force value over time. The equipment is shown in Figure 9 and Figure 10.

The test stand consists of two assemblies. The function of the first assembly is to rigidly clamp one end of the specimen. The second assembly consists of two carriages that hold the other end of the specimen. The drive system is responsible for increasing the cutting force until the metal–polymer bond is broken. The drive assembly uses a bipolar, two-phase stepper motor. The usage of stepper motors allow for very accurate positioning of the rotating shaft position. Controlling the frequency of control impulses allows the rotational speed to be changed with simultaneous control of the executed displacement. By using this type of drive, it is possible to set a precise value of displacement and control the increase in shear force.

**Figure 11:**
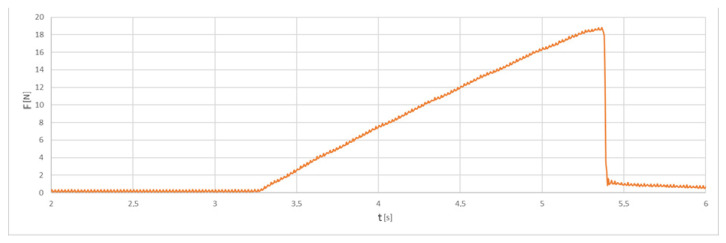
An example of the force trend during the metal–polymer shear strength test.

The tests were carried out with 3 repetitions. During the tensile and shear tests, the maximum force was determined. An example of the recorded shear force values is shown in Figure 11.

The maximum value of the force at which the rupture of the bond occurred is clearly visible in the presented chart. Using the maximum force values, the strength was calculated. The size of the nominal contact area was different for each of the tested samples. The differences resulted from the 3D FDM printing technology, which caused changes in the shape and size of the joint surface. When calculating the rupture stress, the size variation in the contact area was taken into account.

#### 2.7.3. Tribological Studies

The gold standard in tribological research is the use of a pin-on-disc test stand. However, it is not possible to achieve reciprocating motion that mimics the way the materials cooperate in the prosthetic joint on a pin-on-disc stand. In order to ensure the samples had a standardized form, the ASTM G99-17 standard was used. This standard describes testing with the use of a pin-on-disc test stand, although the samples intended for the pin-on-disc test stand are well suited for the test stand used in the tests (described below). The plastic-coated sample had a cylindrical shape (cylinder diameter of 8 mm and height of 10 mm).

During the tribological tests, a 3D-printed cylindrical specimen was pressed with a normal force onto a Ti6Al4V titanium alloy plate. The unit pressure in tribological tests was *p* = 1 MPa. Two types of tests were performed:

- Tests for the coefficient of static friction µ_0_,
- Tests for the coefficient of kinetic friction µ.

**Figure 12:**
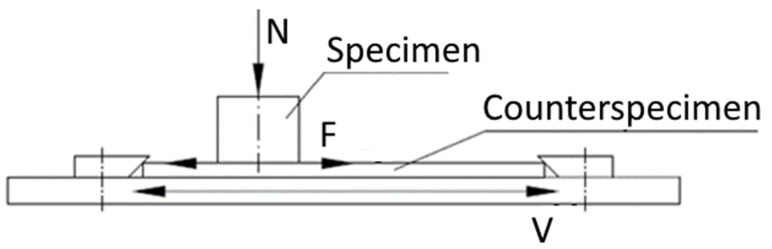
A kinematic scheme of the test stand used for testing friction during the reciprocating motion: N—normal force; F—friction force; V—velocity.

Both types of tests were performed for dry friction and in the presence of water. The friction pair operated dry in the first stage of the test and then water was added to the friction node. The friction force F was recorded at a frequency of 200 Hz. Friction tests were carried out during reciprocating motion. A scheme of the friction pair used during the tests is shown in Figure 12.

The tested friction pair consisted of materials that may occur in the implant with metal–polymer components. Three friction pair were tested:

- PLA–Ti6Al4V;
- PA–Ti6Al4V;
- Ti6Al4V–Ti6Al4V.

**Figure 13:**
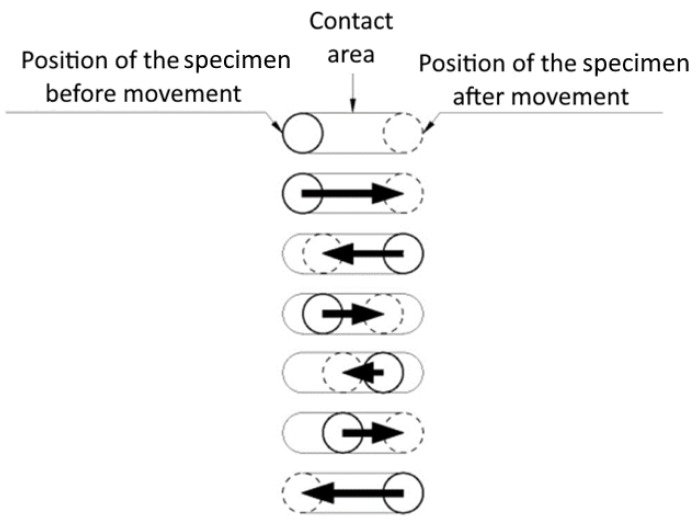
Specimen displacement during static friction measurements.

During static friction coefficient testing, a titanium plate mounted on a moving carriage performed a motion cycle that consisted of 20 motions. During the motion cycle, the test stand performed slow movements (V = 1 mm/s) in both directions. There was a break of 2 s between movements. The distance in each direction was different, which meant the starting point changed for each motion (Figure 13).

**Figure 14:**
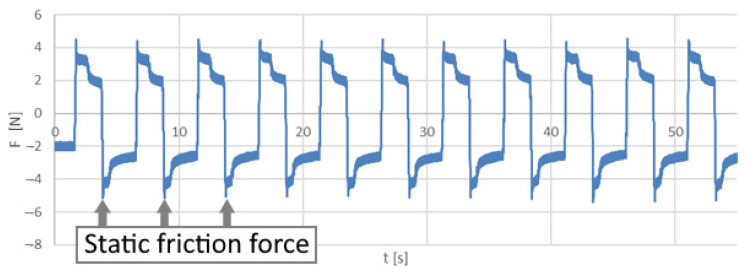
An example of the friction force values recorded during the static friction test.

Elastomeric washers used between the force sensor and the carriage caused the value of the force to increase gradually during the movement. As soon as the friction force reached a value equal to the static friction force the motion occurred. The transition from static to kinetic friction resulted in an immediate decrease in the measured force. The peak value of the friction force was assumed as the static friction force (Figure 14).

After one measurement series, 40 static friction force values were obtained. The mean value from 36 measurements (the 4 most extreme values were rejected) was taken as the static friction force value. The average static friction force values were used to calculate static friction coefficients.

The kinetic friction coefficient tests were performed in reciprocating motion at a sliding velocity of V = 20 mm/s. The friction force was taken as the value after the velocity stabilized.

#### 2.7.4. Microscopic Observations

Titanium alloy–polymer bonds were subjected to microscopic observations. The bonds were checked before detachment and the surface of the polymer sample was observed after detachment. The tests were performed using a Nikon Eclipse MA200 light microscope. The observations were made at magnifications in the range of 100–500x. The images were recorded with a Visitron Systems digital camera with Spot Advanced and NIS Elements BR software. Metallographic specimens were prepared using a grinding and mechanical polishing process.

Microscopic observations were also carried out using a Phenom XL scanning electron microscope at magnifications of 500–2000x. An accelerating voltage range of 5–25 kV was applied during the experiments. Material contrast observations were performed using SE and BSE detectors.

## 3. Results

### 3.1. Results of Strength Tests

The tear and shear rupture stress values are summarized in Table 4.

**Figure 15:**
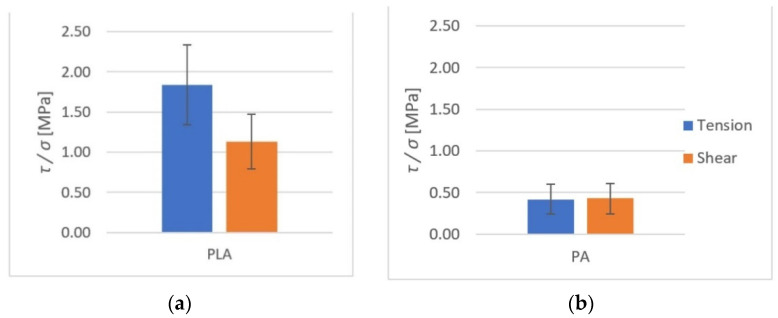
The shear and tensile strengths of the tested metal (Ti6Al4V)–polymer components: (**a**) PLA; (**b**) PA.

**Figure 16:**
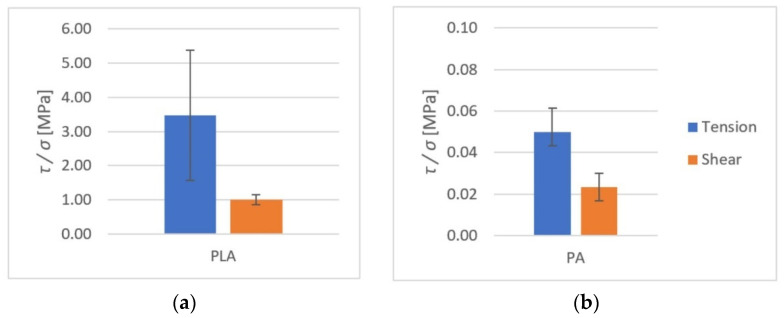
The shear and tensile strengths of the tested metal (Ti6Al4V)–polymer components: (**a**) PLA; (**b**) PA.

The average rupture stress values are presented in Figure 15 and Figure 16.

As it can be observed, higher tensile strength (both at rupture and shear) was seen in components made of PLA. For PLA–Ti6Al4V bonds, the effect of surface roughness on tensile strength was observed. For samples with higher roughness, the tensile strength was almost doubled.

For PA–Ti6Al4V bonds, the rupture strength was very low. Many PA prints detached from Ti6Al4V during transit or after attaching to test equipment holders.

### 3.2. Results of Microscopic Observations

In order to formulate a qualitative assessment of the Ti6Al4V–polymer bonds, microscopic observations of the bonding area were carried out. In addition, the surfaces of polymer samples detached from Ti6Al4V were examined. Microscopic observations of the samples revealed differences in the quality of the metal–polymer bonds.

#### 3.2.1. Ti6Al4V–PLA Bond

**Figure 17:**
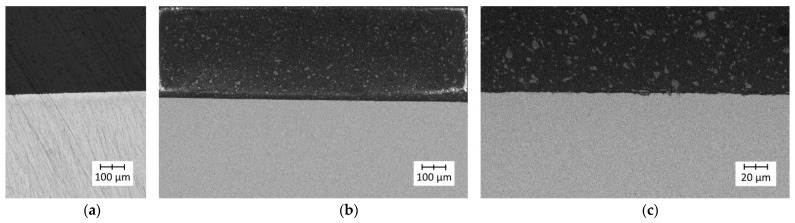
Microscopic images of Ti6Al4V (lower roughness)–PLA bonds: (**a**) light microscopy—a continuous metal–polymer bond was visible; (**b**) SEM (lower magnification)—a high-quality metal–polymer bond was visible; (**c**) SEM (higher magnification)—a high-quality metal–polymer bond was visible.

The images recorded during the analysis of the Ti6Al4V (lower roughness)–PLA bonds are presented in Figure 17.

**Figure 18:**
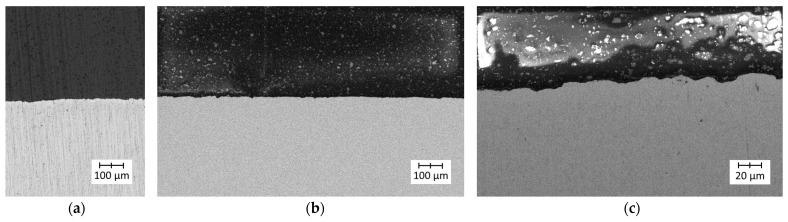
Microscopic images of Ti6Al4V (higher roughness)–PLA bonds: (**a**) light microscopy—a continuous metal–polymer bond was visible; (**b**) SEM (lower magnification)—a high-quality metal–polymer bond was visible; (**c**) SEM (higher magnification)—a high-quality metal–polymer bond was visible.

The images recorded during the analysis of the Ti6Al4V (lower roughness)–PLA bonds are presented in Figure 18.

The microscopic observations of Ti6Al4V–PLA bonds revealed that in both cases (for lower and higher roughness levels of the titanium alloy specimen) the interface was continuous and free from delamination, pores, blisters, or other structural defects. The different surface roughness levels of the metal substrate did not affect the quality of the bonds.

**Figure 19:**
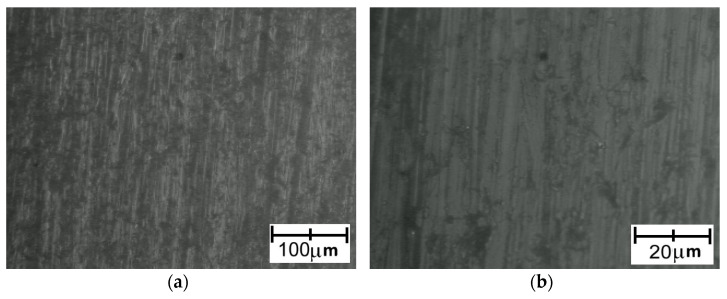
Microscopic images of the PLA surface after detaching from Ti6Al4V titanium alloy with lower roughness: (**a**) low magnification; (**b**) high magnification.

**Figure 20:**
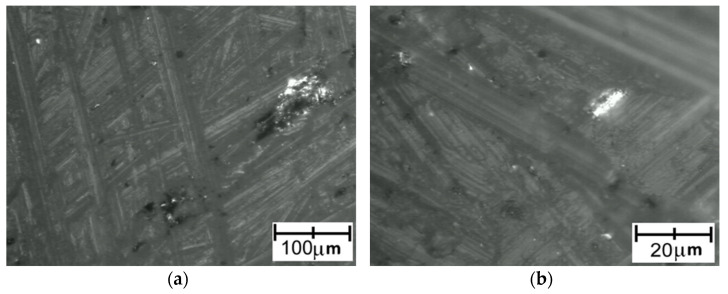
Microscopic images of the PLA surface after detaching from Ti6Al4V titanium alloy with higher roughness: (**a**) low magnification; (**b**) high magnification.

The images recorded for PLA detached from a Ti6Al4V titanium alloy sample with lower roughness are presented in Figure 19. Images recorded for PLA detached from a Ti6Al4V titanium alloy sample with higher roughness are presented in Figure 20.

After detaching the PLA sample, the surface texture seemed to be smoother than for PA. This indicated that the actual adhesion surface was greater for PLA than for PA, which could be responsible for the higher strength of Ti6Al4V–PLA bonds compared to Ti6Al4V–PA bonds.

#### 3.2.2. Ti6Al4V–PA Bond

**Figure 21:**
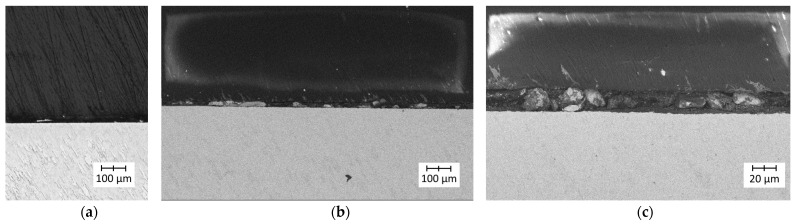
Microscopic images of Ti6Al4V (lower roughness)—PA bonds: (**a**) light microscopy—a continuous metal–polymer bond was visible; (**b**) SEM (lower magnification)—a high-quality metal–polymer bond was visible; (**c**) SEM (higher magnification)—a high-quality metal–polymer bond was visible.

The images recorded during the analysis of the Ti6Al4V (lower roughness)–PA bonds are presented in Figure 21. The Ti6Al4V (higher roughness)–PA bonds were so highly unstable that complete discontinuity of the bonds occurred, meaning this specimen could not be microscopically examined.

In the terms of Ti6Al4V–PA bond, a lack of permanent connection between the polymer and the titanium substrate was observed. Local delamination between the materials was clearly visible.

**Figure 22:**
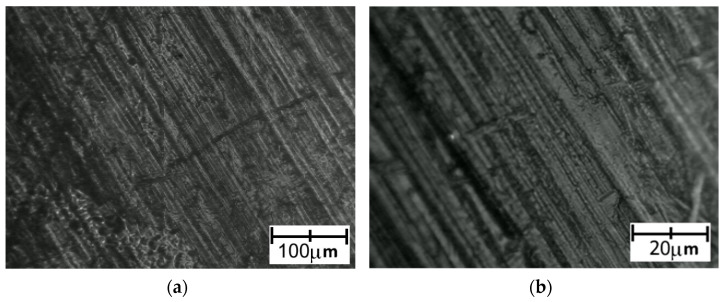
Microscopic images of PA surface after being detached from Ti6Al4V titanium alloy with lower roughness: (**a**) low magnification; (**b**) high magnification.

**Figure 23:**
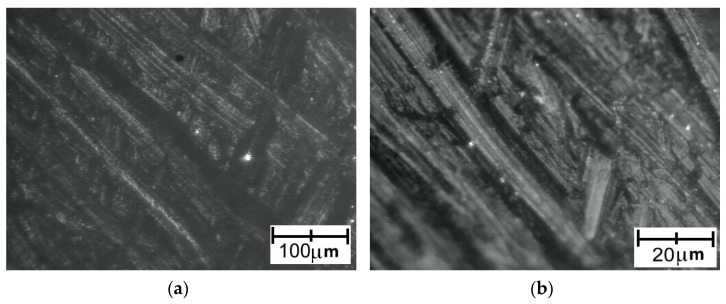
Microscopic images of PA surface after being detached from Ti6Al4V titanium alloy with higher roughness: (**a**) low magnification; (**b**) high magnification.

Images recorded for PA detached from a Ti6Al4V titanium alloy with lower roughness are presented in Figure 22. Images recorded for PA detached from a Ti6Al4V titanium alloy with higher roughness are presented in Figure 23.

Although the surface of the metal component showed increased roughness, the polymer surface texture seemed to be smooth.

### 3.3. Result of Tribological Tests

The results of tribological tests are shown in Table 5.

**Figure 24:**
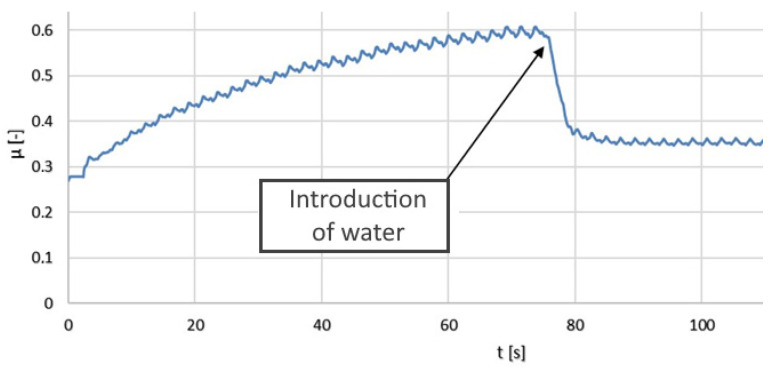
The kinetic friction coefficient µ values for the pair composed of Ti6Al4V and PLA, showing dry friction and friction in the presence of water.

**Figure 25:**
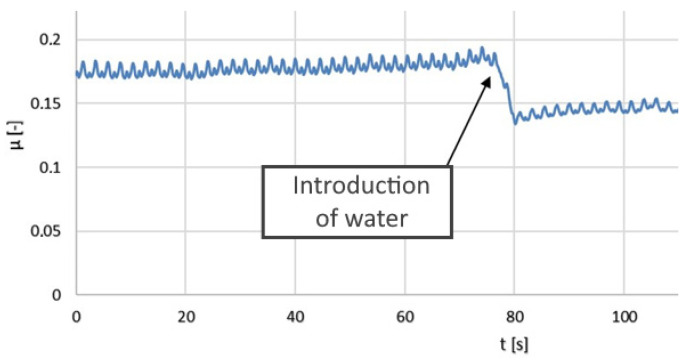
The kinetic friction coefficient µ values for the pair composed of Ti6Al4V and PA, showing dry friction and friction in the presence of water.

**Figure 26:**
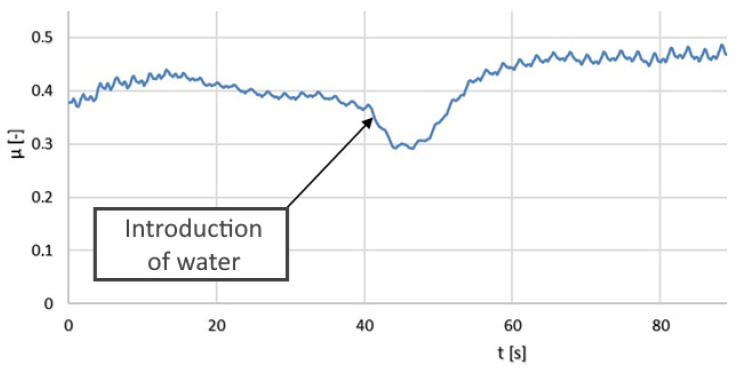
The kinetic friction coefficient µ values for the pair composed of Ti6Al4V and Ti6Al4V, showing dry friction and friction in the presence of water.

The results of the tribological tests are also presented in the form of graphs (moving average) in Figure 24, Figure 25 and Figure 26.

The friction coefficient values for the PLA–Ti6Al4V pair increased with time. A slight seizing effect occurred. When water was applied, the coefficient of friction decreased significantly. Dry operation should not be allowed for this type of friction pair.

In the terms of the PA–Ti6Al4V pair, the friction coefficient values were significantly lower than for the PLA–Ti6Al4V pair. Water had a positive effect on the coefficient of friction. Seizing did not occur for the analyzed friction pair. The friction coefficient was stable and relatively low, which confirmed the good tribological properties of PA.

For the MoM (metal-on-metal) Ti6Al4V–Ti6Al4V pair, the presence of water had a very brief effect. A continuous increase in the resistance to motion was observed. This is typical for pairs consisting of two parts made of the same material. The oxide layer was destroyed, strong adhesive interactions occurred and seizing emerged. The measured kinetic friction coefficient values for this pair were higher than for the Ti6Al4V–PA pair but lower than for the Ti6Al4V–PLA pair.

**Figure 27:**
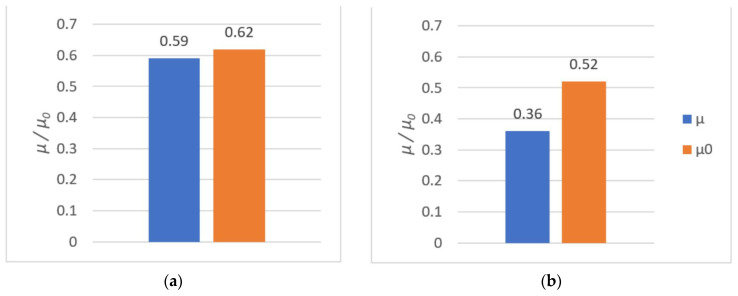
The results of tribological investigations of the PLA–Ti6Al4V pair: (**a**) dry friction; (**b**) friction in the presence of water.

**Figure 28:**
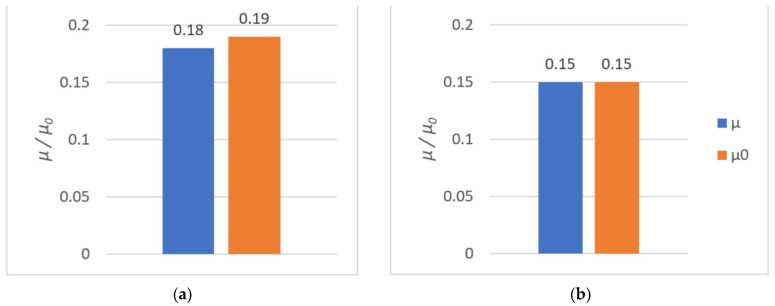
The results of tribological investigations of the PA–Ti6Al4V pair: (**a**) dry friction; (**b**) friction in the presence of water.

**Figure 29:**
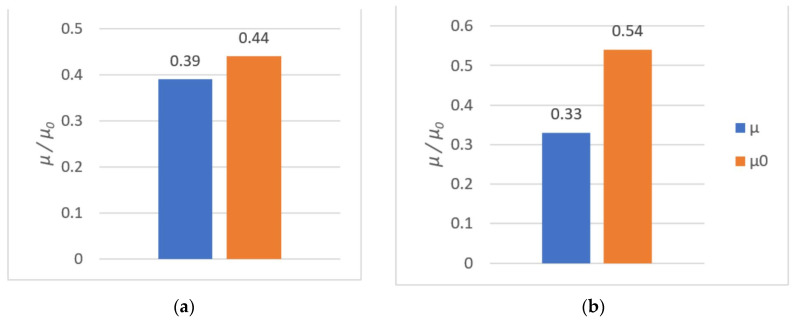
The results of tribological investigations of the Ti6Al4V–Ti6Al4V pair: (**a**) dry friction; (**b**) friction in the presence of water.

All results from both the kinetic and the static friction tests are presented in Figure 27, Figure 28 and Figure 29.

## 4. Discussion

### 4.1. Strenght Tests

Analyzing the results of the tensile and shear strength tests of metal–polymer bonds, it can be concluded that:

- The deposition of the biocompatible material by 3D FDM printing on titanium alloy was possible; however, the rupture strength of the tested bonds was low. The prints made from PLA had a tensile strength range of about 20 to 80 MPa [74,75]. This range was very wide, since the mechanical properties of FDM 3D-printed element depend on numerous printing parameters. The sample printed with the same parameters as in this article had a strength value of 29 MPa. In terms of the 3D FDM-printed PA, the strength was approximately 33 MPa [76,77]. The obtained strength values for metal–polymer bonds did not exceed 2 MPa at tension and 3.5 MPa at shear. The rupture strength of the metal–plastic bond was much lower than the rupture strength of the weaker polymer material;
- The results of the strength tests showed expanded uncertainty (expanded uncertainty is up to 55% of the mean value). This means that the process of printing had low repeatability and the bonds were characterized by different rupture strengths;
- The usage of FDM 3D printing to deposit plastic as a part of the manufacturing process of endoprostheses would require an improvement, since the tensile and shear strength were too low and the repeatability of the printing process was poor;
- Ti6Al4V–PA bonds had 2–4 times lower tensile and shear strengths than Ti6Al4V–PLA;
- The effect of surface roughness on tensile strength was observed only for Ti6Al4V–PLA components. For samples with higher roughness, the tensile strength of the bonds was almost doubled. For shear strength, the effect of roughness was negligible.

The surface free energy value for PLA [78] is similar to that of PA [79], so adhesive interactions with titanium alloy will have similar strengths for both plastics. The parameter that differs between the investigated plastics is the thermal expansion coefficient. This is crucial, since the print shrinks and microslides between plastic and metallic substrates occur during cooling. As a result, the adhesive and mechanical bonds are broken. PLA has a significantly lower thermal expansion coefficient than PA [80,81]. Moreover, PLA needs to be heated to a lower temperature than PA to achieve material flow. As a result, the temperature difference during cooling is smaller for PLA and the shrinkage is also smaller. Therefore, the difference between the shrinkage of Ti6Al4V and PLA is smaller than that of Ti6Al4V and PA. Hence, in terms of Ti6Al4V–PLA, fewer adhesive and mechanical bonds are destroyed. This is another potential explanation for why Ti6Al4V–PA bonds have significantly lower tensile and shear strengths than Ti6Al4V–PLA bonds.

The higher strength of Ti6Al4V–PLA may also be a result of a larger actual contact area. PLA likely has the ability to fill surface irregularities better. Here, PA did not fill the irregularities as well, and as a result the strength of the mechanical bonds was lower and adhesive bonds were formed only on the tops of irregularities. During the tests of PA–Ti6Al4V components, no effect of surface roughness on the bond strength was observed, which may also confirm that at higher roughness levels the irregularities are not filled and the effect of increased roughness is not visible. The melt mass–flow rate (MFR) for PLA was 14.48 g/10 min (load = 2.16 kg, temperature = 190 °C) [65] and for PA was 9.23 g/10 min (load = 5.00 kg, temperature = 235 °C) [66]. This means that PLA is more fluid and easier to process by extrusion. The greater fluidity of the material probably caused the better filling of irregularities.

Additionally. 3D FDM prints differ significantly from elements made using the conventional injection method due to their layered structure [82]. The destruction of a 3D FDM print starts with damage between the layers [69]. The weak points of 3D prints are the defects between the layers, namely the voids, air gaps and points of stress concentration [83,84,85]. Microscopic observations of the PA–Ti6Al4V bonds showed that local delamination between the materials was clearly visible. It is very possible that the abovementioned defects also appear at the metal–plastic interface and reduce the strength of the bond. When improving the method used for depositing plastics onto metals, special attention ought to be paid to preventing such defects.

### 4.2. Tribological Tests

The tribological tests allowed us to evaluate the sliding properties of materials intended for 3D printing of metal–polymer components for implants. After analyzing the obtained values of coefficient of friction, it was concluded that:

- The friction pair Ti6Al4V–PA had the lowest coefficient of friction. The coefficient of friction values were low at 0.15 in terms of dry friction and 0.18 in presence of water. Seizure was not observed during the use of these materials. One of the most frequently used pairs in endoprostheses is an UHMWPE cup with a Ti6Al4V head. For such pairs, the coefficient of friction (dry friction) is in the range of 0.07–0.14 [86]. Hence, the coefficient of friction value of 0.15 for Ti6Al4V–PA was very good;
- The PLA–Ti6Al4V pair showed a significant coefficient of friction value of µ = 0.59. Therefore, PLA is not suitable for cooperation with titanium alloy in endoprostheses. PLA could be used as a material for scaffolds printed directly onto titanium alloy. Such scaffolds could be introduced in selected regions of the endoprosthesis to support tissue growth. Moreover, due to the biodegradability of PLA, the scaffold could be later removed from the patient’s body. The FDM method is well suited for creating scaffolds [87];
- The presence of water lowered the coefficient of friction values for most of the tested friction pairs. The Ti6Al4V–Ti6Al4V pair was an exception, since the coefficient of static friction in the presence of water was highest (µ 0 = 0.54);
- Ti6Al4V–PLA and Ti6Al4V–Ti6Al4V pairs had a tendency to seize. The addition of water reduced the coefficient of friction, although this effect was short-lived.

The authors of [35] proved that specimens 3D-printed from PCU had high porosity and absorbed lubricants better. Therefore, they acted similarly to the natural lubrication mechanism of the meniscus. In terms of the cooperation of PLA with Ti6Al4V, this kind of effect was not observed. The addition of a lubricant (water) caused that pair to seize. In terms of the Ti6Al4V–PA pair, the friction coefficient decreased by about 17% after the addition of water, and this effect persisted over time. Moreover, porosity in 3D FDM-printed PA specimens [88] is significantly lower than in PLA specimens [89]. The effect associated with porosity is probably not only caused by material processing by 3D FDM printing, but also likely depends on the material used. The porosity of the 3D FDM prints can be reduced or increased, e.g., by using other printing parameters [90]. While carrying out further work on the influence of pores on the tribological properties of 3D prints, it is worth testing samples from the same material but with different porosity levels.

## 5. Conclusions

This research showed that the deposition of PLA and PA by FDM 3D printing directly on Ti6Al4V titanium alloy is possible. However, the usage of FDM 3D printing to deposit plastic as a part of the manufacturing process of endoprostheses would require improvements, since the tensile and shear strength are too low and the repeatability of the printing process is poor. The benefits of the application of this method are so impressive that it is worth continuing work on this issue. Further research ought to be focused on improving the method of plastic deposition and attempts to use other materials.

## Figures and Tables

**Table 1:** Properties of Zortrax filaments used in research [65,66].

Properties	Test Method	Z-PLA	Z-Nylon
Tensile Strength	ISO 527:1998	47.95 MPa	33.22 Mpa
Breaking Stress	ISO 527:1998	46.53 Mpa	29.81 Mpa
Elongation at Max Tensile Stress	ISO 527:1998	3.80%	9.17%
Elongation at Break	ISO 527:1998	4.32%	23.62%
Bending Stress	ISO 178:2011	56.80 Mpa	38.30 Mpa
Flexural Modulus	ISO 178:2011	1.47 Gpa	781.01 Mpa
Izod Impact, Notched	ISO 180:2004	3.14 kJ/m^2^	12.81 kJ/m^2^
Melt Flow Rate	ISO 1133:2006	14.48 g/10 min (Load 2.16 kg, Temperature 190 °C)	9.23 g/10 min (Load 5.00 kg Temperature 235 °C)
Specific Density	ISO 1183-3:2003	1.292 g/cm^3^	1.027 g/cm^3^
Shore Hardness (D)	ISO 868:1998	79.8	62.0

**Table 2:** Roughness parameters for surfaces of selected Ti6Al4V alloy samples.

Roughness Parameter	Value for the Sample with Lower Roughness (µm)	Value for the Sample with Higher Roughness (µm)
R_a_	0.37	6.2
R_z_	2.6	36.5

**Table 3:** Manufacturing parameters for 3D-printed samples.

Deposited Plastic	PLA	PA
Metal on which plastic is deposited	Ti6Al4V titanium alloy	
Metal temperature during deposition of the first three layers of plastic	220 °C	260 °C
Metal temperature during deposition of the fourth and subsequent layers of plastic	60 °C	160 °C
Temperature of the deposited plastic	220 °C	260 °C
3D printing nozzle diameter	0.4 mm	
Layer height	0.2 mm	
Infill density and infill pattern	90% (the infill lines printed at a 45° angle)	
First layer print speed	Reduced (50%)	
First layer flow ratio	Normal (100%)	
First layer density	Normal (100%)	

**Table 4:** Rupture stress values of the tested Ti6Al4V–polymer bonds.

Material	Type of Load	Stress at Rupture of the Bond [MPa]	
		Metal Specimen with Lower Roughness	Metal Specimen with Higher Roughness
PLA	Tension	1.84 ± 0.50	3.47 ± 1.91
	Shear	1.13 ± 0.34	1.00 ± 0.14
PA	Tension	0.42 ± 0.18	0.05 ± 0.01
	Shear	0.43 ± 0.18	0.02 ± 0.01

**Table 5:** Static and kinetic friction coefficients.

Tribological Pair	Dry Friction		Friction in the Presence of Water	
	µ	µ_0_	µ	µ_0_
PLA–Ti6Al4V	0.59	0.62	0.36	0.52
PA–Ti6Al4V	0.18	0.19	0.15	0.15
Ti6Al4V–Ti6Al4V	0.39	0.44	0.33	0.54

## Data Availability

The data presented in this study are available on request from the corresponding author.
